# Supplementary material for: Crawling and Gliding: A Computational Model for Shape-Driven Cell Migration
Source: PLoS Comput Biol. 2015 Oct 21;11(10):e1004280. doi: 10.1371/journal.pcbi.1004280 (PMC4619082; doi:10.1371/journal.pcbi.1004280)
Supplement: S1 Code — (ZIP) [file pcbi.1004280.s012.zip › release/tst/doc/html/conrec_8h.html]

Tissue Simulation Toolkit: conrec.h File Reference


Tissue Simulation Toolkit
 0.1.4.1


- Main Page
- Namespaces
- Classes
- Files

- File List
- File Members

Functions

conrec.h File Reference

This graph shows which files directly or indirectly include this file:

Go to the source code of this file.

|  |  |
| --- | --- |
| Functions | |
| int | conrec (double \*\*d, int ilb, int iub, int jlb, int jub, double \*x, double \*y, int nc, double \*z, Graphics \*g, int colour=1) |
|  | Paul Bourke's conrec algorithm to draw contour lines of PDE fields. More... |
|  | |

## Function Documentation

|  |  |  |  |
| --- | --- | --- | --- |
| int conrec | ( | double \*\* | *d*, |
|  |  | int | *ilb*, |
|  |  | int | *iub*, |
|  |  | int | *jlb*, |
|  |  | int | *jub*, |
|  |  | double \* | *x*, |
|  |  | double \* | *y*, |
|  |  | int | *nc*, |
|  |  | double \* | *z*, |
|  |  | Graphics \* | *g*, |
|  |  | int | *colour* = `1` |
|  | ) |  |  |

Paul Bourke's conrec algorithm to draw contour lines of PDE fields.

C-code Copyright (c) 1996-1997 Nicholas Yue.

References max, min, Graphics::Point(), xsect, and ysect.

Referenced by PDE::ContourPlot().


---

Generated on Thu Aug 14 2014 22:04:01 for Tissue Simulation Toolkit by  

 1.8.6
